# Direct Printing of 1-D and 2-D Electronically Conductive Structures by Molten Lead-Free Solder

**DOI:** 10.3390/ma10010001

**Published:** 2016-12-22

**Authors:** Chien-Hsun Wang, Ho-Lin Tsai, Weng-Sing Hwang

**Affiliations:** Department of Materials Science and Engineering, Research Center for Energy Technology and Strategy, National Cheng-Kung University, No. 1, Ta-Hsueh Road, Tainan 70101, Taiwan; terence0910@gmail.com (C.-H.W.); hltsai0722@gmail.com (H.-L.T.)

**Keywords:** lead-free solder, inkjet printing, micro patterns, micro droplet, metallic line

## Abstract

This study aims to determine the effects of appropriate experimental parameters on the thermophysical properties of molten micro droplets, Sn-3Ag-0.5Cu solder balls with an average droplet diameter of 50 μm were prepared. The inkjet printing parameters of the molten micro droplets, such as the dot spacing, stage velocity and sample temperature, were optimized in the 1D and 2D printing of metallic microstructures. The impact and mergence of molten micro droplets were observed with a high-speed digital camera. The line width of each sample was then calculated using a formula over a temperature range of 30 to 70 °C. The results showed that a metallic line with a width of 55 μm can be successfully printed with dot spacing (50 μm) and the stage velocity (50 mm∙s^−1^) at the substrate temperature of 30 °C. The experimental results revealed that the height (from 0.63 to 0.58) and solidification contact angle (from 72° to 56°) of the metallic micro droplets decreased as the temperature of the sample increased from 30 to 70 °C. High-speed digital camera (HSDC) observations showed that the quality of the 3D micro patterns improved significantly when the droplets were deposited at 70 °C.

## 1. Introduction

Microelectromechanical system (MEMS) technologies are widely used for many applications. However, inkjet printing (IJP) technology is an attractive alternative to MEMS, due to its low-cost, rapid processing, and accuracy [1,2]. Electrical components have recently been produced for the electronics packaging industry using inkjet printing [3,4,5]. With this, metal micro-droplets can be printed directly, and this has thus become one of the most attractive processing methods in MEMS [6,7,8,9,10,11,12]. For instance, molten tin (Sn) micro droplets have been used to demonstrate the sensing capability with a microdrop generator [13]. The solder-bump of flip chip packages offers a number of advantages, including high input/output connects, cost, high-frequency performance, and easy assembly [14]. The electrical interconnections produced by microdeposition of metal alloys can be ink-jet printed in simple line and dot patterns [15]. The through-silicon via (TSV) by the molten solder ejection method (MSEM) has also been carried out to produce high frequency interconnections for radio frequency-microelectromechanical system (RF-MEMS) switches [16,17].

While a great deal of effort has been made on the deposition and solidification of molten droplets in the solder jet bumping process, little is known about the coalescence of molten micro droplets in relation to the micro line structure of inkjet printing. Tsai et al. [18] examined the effects of a bipolar pulse waveform on the droplet formation behavior, and their experimental results showed that molten lead-free solder can be jetted into single droplets under a suitable nitrogen back-pressure of between 10 and 21 kPa. Li et al. [19] discussed the successive deposition of molten Al droplets on a horizontally moving substrate under different substrate velocities, and showed that the substrate velocities are affected by the solidification times of the droplets deposited on the horizontally moving substrate. Luo et al. [20] investigated the solidification of solder droplets on a substrate with different temperatures in relation to solder bump formation. The results showed that the height of bump droplets decreases when the substrate temperature increases. Ristenpart et al. [21] investigated the coalescence dynamics of two droplets on a highly wettable substrate. They have found that the width of the growing meniscus bridge between the two droplets exhibited power-law behavior. Lass et al. [7] developed a process to enhance metal micro droplet generation by pneumatic actuation based on the StarJet method. Based on the structures generated in these works, it is clear that the printing parameters and printed structures can be controlled based on the droplet temperature.

The mergence of molten metal droplets on the substrate is important due to the overlap between the droplets during deposition. Although a large number of experimental and simulation [18,19,20,22,23] studies have been made on metal droplets, little is known about the mergence of molten metal droplets using a high-speed digital camera. Li et al. [19] and Li et al. [23] reported that the molten Al droplets have vertical direction and L-shaped ridges on the surface of droplets. These results indicate that the molten Al droplets solidified in a layer by layer mode due to the high heat conductivity. Therefore, in the present case, molten metal droplets were successfully deposited in a metallic line using an inkjet printing method under different substrate velocities and temperatures. The definitions of the dimensionless numbers (Weber, Reynolds, Ohnesorge, and Stefan number) used in this work are as follows [24,25,26,27,28,29]

(1) $$\mathrm{We}=\frac{{\rho D}_{0}\;\nu^{2}}{\sigma },$$

(2) Re=ρD0 νη,

(3) $$\mathrm{Oh}=\frac{\eta }{\sqrt{D{}_{0}\;\sigma \rho }},$$

(4) $$\mathrm{Ste}=C_{p}\frac{T_{m}-T_{s}}{L},$$

where ν, ρ and σ are the impact velocity, density and surface tension of the droplet, respectively. Furthermore, η is the droplet’s dynamic viscosity, C_p_ is the specific heat of the liquid, T_m_ is the equilibrium freezing temperature of solder, T_s_ is the substrate temperature of the solder, L is the latent heat of fusion and D_0_ is the initial diameter before impact.

The formation of metallic lines via continuous droplet deposition on a substrate is discussed from three perspectives. First, the droplets must be deposited on the substrate. Second, the metallic line of the droplet-substrate interface will become larger than the initial droplet diameter. Finally, the width and height uniformity of deposited droplet on the surface of the substrate is an important factor in the printing of metal lines. Li et al. [19] found that the solidification fronts are always parallel to the contact lines between droplets, and advance within the liquid of the droplet, moving vertically to the contact lines.

The present paper aims to improve the coalescence of micro metal droplets by using an impacting inkjet printing system, which will be utilized as a droplet generator for most of the experimental work of this study. The major goal is to observe the widths and shapes of the molten micro droplets, and to determine the accurate print quality in order to obtain successive mergence of molten droplets using the drop-on-demand (DOD) inkjet printing method. The effects of the dot spacing, sample temperature and variations in motion velocity of consistent droplets on the shapes of the conducting lines were recorded. The results of this work may provide useful information to achieve the appropriate mergence of solder for the fabrication of micro line structures.

## 2. Experimental Methods

### 2.1. Apparatus and Method Description

**Figure 1:**
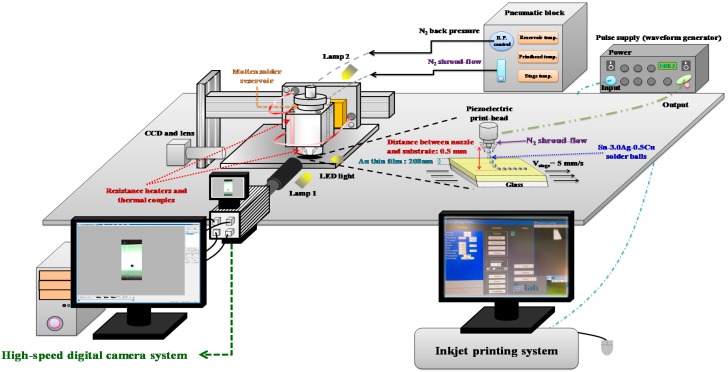
Schematic diagram of the experimental apparatus for the molten metal deposition and mergence.

Figure 1 shows a schematic diagram of the experimental apparatus for the molten metal deposition and mergence. The experiments were performed using a DOD inkjet printing system (MicroFab Technologies Inc., JetLab4, Plano, TX, USA), which consists of different pneumatic, heating, printing and monitoring blocks. The pneumatic block provides nitrogen (N2) to alter the back-pressure of the molten solder reservoir, and could also be used to regulate the N2 shroud-flow around the piezoelectric print head to prevent the solder and nozzle from oxidizing until the jet apparatus has cooled to room temperature during the experiment. The heating block heats the solder reservoir and the piezoelectric print-head for ejecting ink up to 230 °C, and the solder is completely melted with a thermal device. The printing block is composed of a piezoelectric print-head (MJ-SF-04-50-8MX, MicroFab Technologies Inc.) and a pulse supply (waveform generator). The diameter of the piezoelectric print-head orifice is 50 μm. The pulse supply provides a bipolar pulse waveform of varying pulse time and voltage. The monitoring block is used to observe and record the deposition of droplets at a high frequency (up to 198,000 fps) with a good resolution, and includes a high-speed digital camera (HSDC) system (Memrecam GX-3, NAC Image Technology, Tokyo, Japan) with a high-speed digital camera lens and two halogen lamps. There are 52 × 80 pixels recorded at 100,000 frames per second on the HSDC used in this study.

The morphology of the solidified drops was observed by scanning electron microscopy (FE-SEM, JSM-7001F, JEOL, Tokyo, Japan). This thermal field emission electron gun (FE-SEM) operates at 10 kV, and then the analysis of the solidified droplets was carried out at magnifications of 200×, 600× and 1000×. Energy dispersive X-ray (EDX, AMETEK, Inc., Berwyn, PA, USA) spectrometry analyses were also performed to examine the elemental composition at selected areas.

### 2.2. Materials and Sample Preparation

Commercial lead-free solder of Sn-3 wt % Ag-0.5 wt % Cu was employed as the ink material in this study. The liquidus and the solidus temperatures were 221 and 216 °C, respectively. There is increasing awareness of the threats to both human health and the environment due to lead. The objectives of the Waste Electrical and Electronic Equipment (WEEE) and Restriction of Hazardous Substances (RoHS) directives were to eliminate lead from electronic products, and these adopted in July 2006 by the European Union [30]. Lead-free solder electronic products are attracting more attention and development in the electronic marketing. Sn-Ag-Cu lead-free solder alloy systems have obtained the most widespread acceptance as a replacement for the conventional Sn-Pb eutectic solder [31,32]. Sn-Ag-Cu alloys are widely used as lead-free solutions for interconnection materials in most areas of the microelectronic packaging industry due to their mechanical and physical properties [33,34,35,36,37,38]. The thermal and physical properties of Sn-3Ag-0.5Cu are shown in Table 1 [18,39]. The gold-plated glass substrates were prepared by sputter deposition from a 99.99% Au target using a Cressington 108 auto sputter coater under vacuum (20 torr) conditions. The deposition current was 40 mA, and the films were grown on glass substrates by sputtering at room temperature with a deposition time of 600 s. The microstructures of the films with a thickness of 208 nm were measured by SEM.

### 2.3. Inkjet Printing Conditions

**Figure 2:**
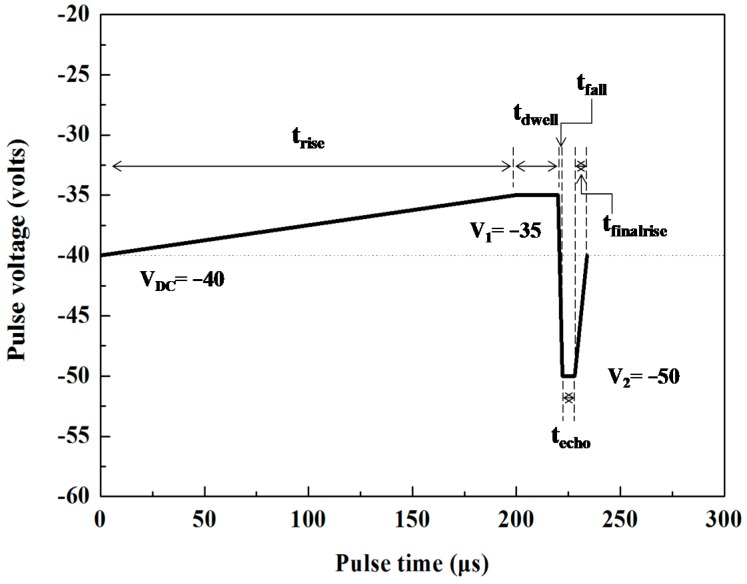
Schematic illustration of the bipolar waveform experimental parameters.

In the DOD inkjet printing method, the molten metal droplets were driven by the bipolar waveform experimental conditions shown in Figure 2. The bipolar waveform is set to be 200 μs for t_rise_, 20 μs for t_dwell_, 2 μs for t_fall_, 6 μs for t_echo_, and 6 μs for t_finalrise_. The waveform variables of the molten metal droplets employed in this study are shown in Table 2. Based on Tsai et al.’s [18] and Wang’s et al.’s [6] findings, the range of the negative pulse voltage for stable droplet formation was between −10 and −50 V. Therefore, the pulse voltages of this experiment were set as V_1_ of −35 V, V_DC_ of −40 V and V_2_ of −50 V, respectively. During the molten metal jet process, a shroud-flow with a nitrogen assist gas jet near the orifice of the piezoelectric print-head was pumped at 1.5 L∙min^−1^, for which a back-pressure of 1.0 kPa in the reservoir was sufficient to maintain a stable phase at different sample temperatures. In order to deposit single droplets, they can be observed with a charge-coupled device (CCD) camera, which includes a microscope objective lens and a light emitting diode (LED). The results show that the regularity and repeatability of droplets are satisfactory when the frequency of the light emitting diode was set as 400 Hz. The jet height of the printing head was fixed at 0.5 mm. The frequency of the droplet ejection (f) can be calculated by [40]

(5) $$p=\frac{V_{\mathrm{stage}}}{f},$$

where *p* is the dot spacing of the droplet, and V_stage_ is the stage velocity.

The parameters used in the experiments are the dot spacing of the droplets, the motion velocity of the stage and the sample temperature. The dot spacing of the droplets and time interval could be adjusted independently by altering the stage velocity and droplet ejection frequency. The droplet ejection frequency was varied from 50 to 1000 Hz.

## 3. Results and Discussion

### 3.1. Effects of Dot Spacing on Droplet Formation and Deposition

**Figure 3:**
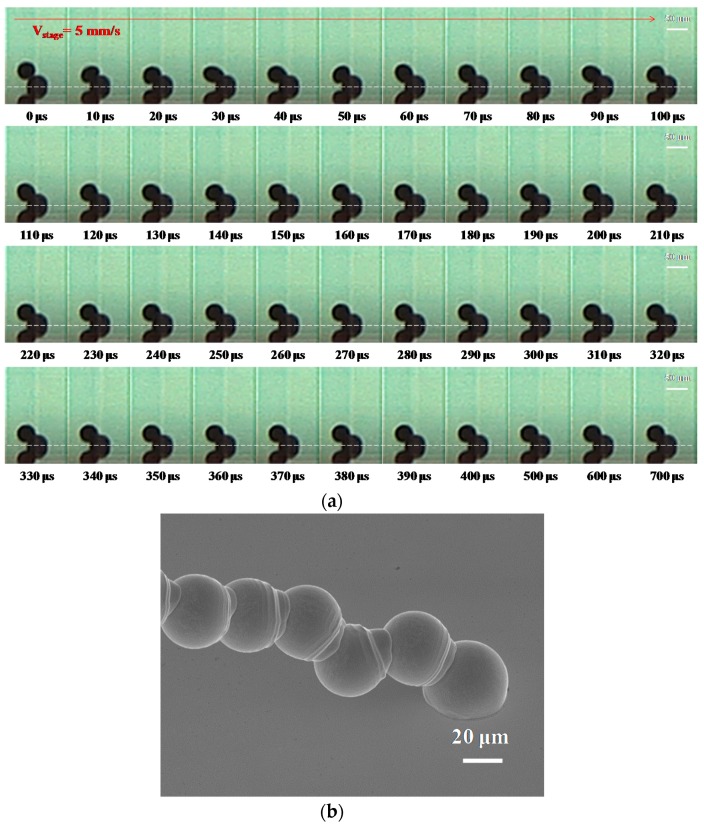
The deposition of molten metal droplets under a dot spacing of 25 μm with the following initial characteristics: diameter D_0_ = 50 μm, velocity = 1.1 m∙s^−1^, stage velocity = 5 mm∙s^−1^ (frequency = 200 Hz) and sample temperature 30 °C (Stefan number Ste = 0.722). (**a**) Evolution of a droplet impacting onto a solidified droplet recorded using a high-speed digital camera with condition A in Table 2. The dashed lines separate the outline of the droplet from that of its reflection on the substrate. The scale bars are 50 μm; (**b**) tilted 40° SEM photograph of the droplets deposited on a gold-plated glass substrate. The scale bar is 20 μm.

Figure 3 shows the deposition process of different dot spacings with condition A (T_rise_ = 200 μs, T_dwell_ = 20 μs, T_fall_ = 2 μs, T_echo_ = 6 μs, T_finalrise_ = 6 μs, V_1_ = −30 V, V_DC_ = −40 V, V_2_ = −50 V and jet height = 0.5 mm), as reported in Table 2. A 50 μm initial diameter (D_0_) solder alloy Sn-3Ag-0.5Cu droplet with 1.1 m∙s^−1^ velocity impacted onto a gold-plated glass substrate at an initial temperature of 30 °C. The parameters mentioned above were applied and calculated by using the dimensionless numbers, and therefore the Weber number We = 0.92, Reynolds number Re = 206.25, Ohnesorge number Oh = 4.7 × 10^−3^. The initial temperature of the molten micro droplet was 230 °C, and the melting point of the solder alloy (Sn-3Ag-0.5Cu) was about 217 °C. The average thickness of the gold-plated substrate was 208 nm. The effects of dot spacing on droplet deposition formation process were first investigated. Figure 3a shows the dot spacing of 25 μm at the parameter corresponding to condition A in Table 2. The stage velocity is also referred to as the droplet ejection frequency, where the sample temperature was fixed at 30 °C. The stage velocity was 5 mm∙s^−1^, and the droplet ejection frequency was 200 Hz. The HSDC is captured at 100,000 frames per second in this study. At 10 μs, the incoming droplet is deposited on the surface of the first (solidified) droplet. In the period 20–160 μs, the micro pillar of molten metal droplets formed and tilted to one side, as the dot spacing was close between droplets. After 170 μs, the incoming (second) droplet moved progressively with time until its static state. It can be clearly seen that the micro pillar structure piled up due to the deposition of molten metal droplets with close spacing. Therefore, we attempted to increase the spacing with a view to more closely linking and joining the dots. Figure 3b shows the droplet deposition on the substrate with the dot spacing of 25 μm, and it can be seen that there was a tilted pile up on the substrate. These results show that with a dot spacing of 25 μm the solder was not deposited on the substrate.

**Figure 4:**
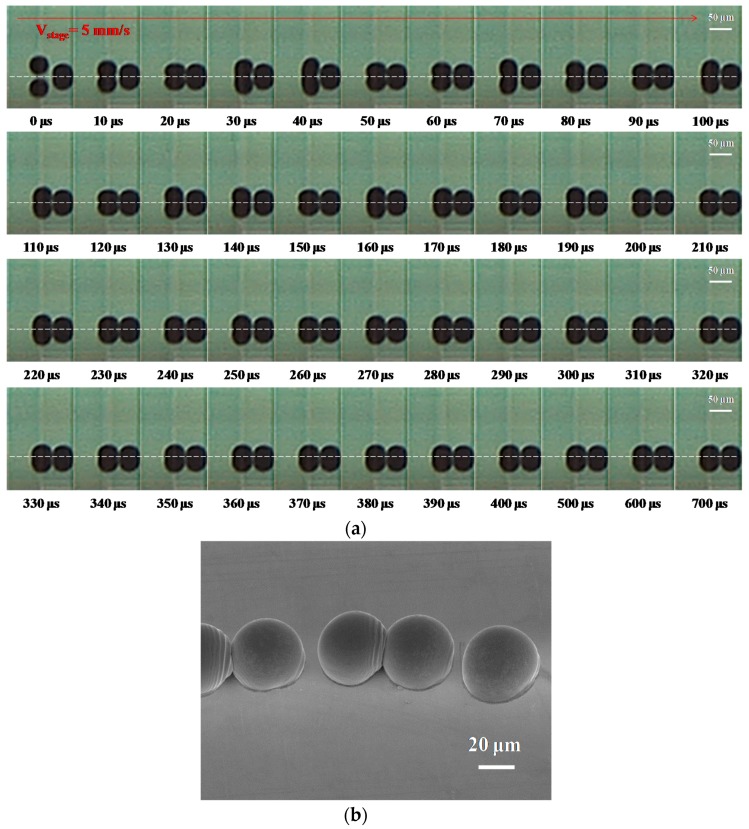
The deposition of molten metal droplets under a dot-spacing of 50 μm with the following initial characteristics: diameter D_0_ = 50 μm, velocity = 1.1 m∙s^−1^, stage velocity = 5 mm∙s^−1^ (frequency = 100 Hz) and sample temperature 30 °C (Stefan number Ste = 0.722). (**a**) Evolution of droplet impacting onto a gold-plated glass substrate recorded using a high-speed digital camera with condition A in Table 2. The dashed lines separate the outline of the droplet from that of its reflection on the substrate. The scale bars are 50 μm; (**b**) tilted 40° SEM photograph of the droplet deposited on a gold-plated glass substrate. The scale bar is 20 μm.

Figure 4a shows the evolution of the dot spacing of 50 μm, with the molten metal droplets deposited on a gold-plated glass substrate with condition B in Table 2. At 10 μs, the incoming (second) droplet is impacted on the side of the first (solidified) droplet. Between 20 and 350 μs, the incoming (second) droplet started post-spreading oscillations and recoiled with time on the substrate, then the droplets will start to solidify with the increasing time. The deposition of the line did not occur when dot spacing of 50 μm was used. The results show that some droplets could be improved when the dot spacing of the droplet increased with a suitable distance and were close to each other on the substrate’s surface. Moreover, the linking and joining of the dots are sensitive to variations in the motion velocity. Suitable pretreatments were thus needed with regard to the effects of motion velocity on the molten metal droplet of Sn-3Ag-0.5Cu alloy. Figure 4b shows an SEM image of the droplet deposition on a gold-plated substrate with the dot spacing of 50 μm. It can be seen that the droplets impacted in different positions and formed an irregular deposit on the substrate. Wang et al. [6] examined the successive deposition behavior of molten micro droplets on a gold-plated glass substrate with inkjet printing techniques, and the molten droplets (with a diameter of 48 μm) achieved complete solidification in a few microseconds (from 220 to 350 μs). The grouping of the two droplets is at least partly related to the process of liquid flow that occurs from the impacting of droplets to solidification. The main reason is that the contact region of the molten micro droplet impacted on the side of the solidified droplet, and the results show that the molten droplet was stuck and then pulled forward to the solidified droplet [19]. Therefore, a solidified droplet means that the molten solder cooled to the temperature of the substrate between 220 and 350 μs. The oscillation tended to decrease rapidly, and alternately spread and recoiled the liquid when the solidification front moved upward. Meanwhile, the remaining liquid volume of the molten micro droplet decreased and solidified. Therefore, the captured images show the two droplets being grouped together. The purpose is to make sure that the subsequently deposited metal droplets will form metallic wires.

**Figure 5:**
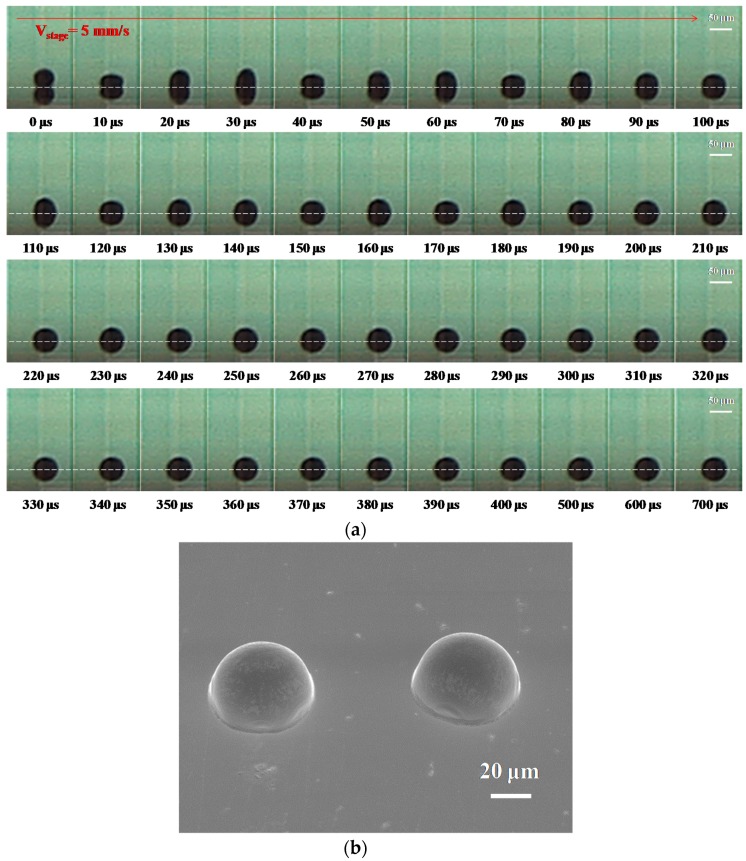
The deposition of a molten metal droplet under a dot spacing of 100 μm with the following initial characteristics: diameter D_0_ = 50 μm, velocity = 1.1 m∙s^−1^, stage velocity = 5 mm∙s^−1^ (frequency = 50 Hz) and sample temperature 30 °C (Stefan number Ste = 0.722). (**a**) Evolution of a droplet impacting onto a gold-plated glass substrate recorded using a high-speed digital camera with condition A in Table 2. The dashed lines separate the outline of the droplet from that of its reflection on the substrate. The scale bars are 50 μm; (**b**) tilted 40° SEM photograph of the droplet deposited on a gold-plated glass substrate. The scale bar is 20 μm.

The deposition of molten metal droplets under a dot spacing of 100 μm was examined by using a high-speed digital camera with condition C in Table 2, as shown in Figure 5a. The stage velocity was 5 mm∙s^−1^, and the droplet ejection frequency was 50 Hz. The incoming droplet was deposited onto a gold-plated glass substrate. It is clear that there was no successive merging between the droplets on the substrate. Figure 5b shows the SEM photograph of the droplet deposited on a gold-plated glass substrate. The results show that the droplets deposited on the substrate had a dot-spacing of approximately 100 μm. Li et al. [19] reported that droplets formed a line along a certain direction with an appropriate substrate velocity and dot spacing. Therefore, we tried to change the motion velocity of the stage and observed the results.

### 3.2. Effects of Motion Velocity on Droplet Formation and Deposition

**Figure 6:**
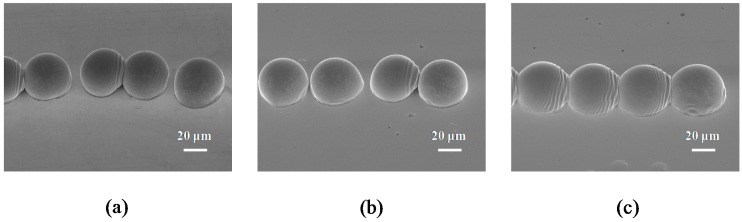
The deposition of a molten metal droplet under different stage velocities with the following initial characteristics: diameter D_0_ = 50 μm, velocity = 1.1 m∙s^−1^, dot spacing = 50 μm, and sample temperature 30 °C (Stefan number Ste = 0.722). (**a**) 5 mm∙s^−1^ (frequency = 100 Hz); (**b**) 10 mm∙s^−1^ (frequency = 200 Hz); (**c**) 50 mm∙s^−1^ (frequency = 1000 Hz). The scale bars are 20 μm.

When the dot spacing of the droplet was fixed at 50 μm. The velocities of the stage were set as 5, 10 and 50 mm∙s^−1^. The frequency of droplet ejection was set at 100, 200 and 1000 Hz, and the time intervals between the droplets were 10, 5 and 1 ms, respectively. Figure 6a,b show the impact and deposition of droplets under different motion velocities of the stage with condition D. The time interval between the droplets was too long when the printing head moved with the stage, resulting in complete solidification. It can be seen that some of the droplets do not link and join the dots when the motion velocity is less than 50 mm∙s^−1^, and thus are deposited separately on the substrate. Figure 6c shows the pattern evolution of the molten micro droplets regularly confined in a 1D metallic line with a width of 55 μm. In the initial stage, the first droplet was observed to form a steady deposit. It is quite obvious that the droplet has stuck and deposited on the substrate. Afterward, the incoming droplet (the second one) is integrated into the top left surface of the first, and then it increases along with the motion velocity until a metallic line with a width of 55 μm can be successfully printed. Images from the experiment show good regular agreement with the SEM photographs.

**Figure 7:**
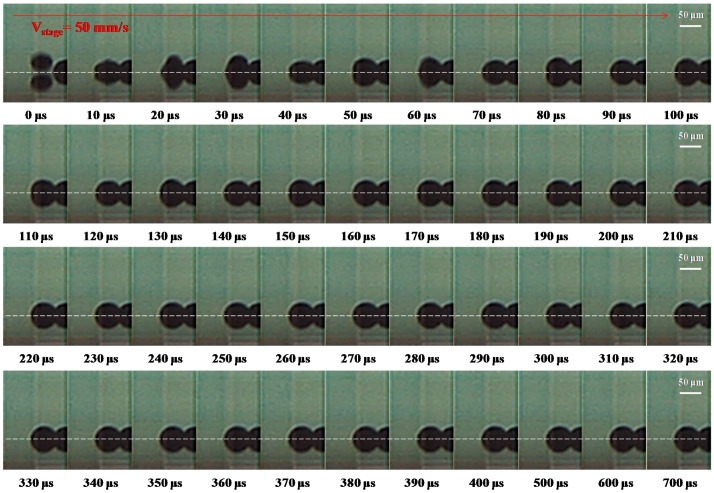
The deposition of a molten metal droplet under a stage velocity of 50 mm∙s^−1^ (frequency = 1000 Hz) with the following initial characteristics: diameter D_0_ = 50 μm, velocity = 1.1 m∙s^−1^, dot spacing = 50 μm, and sample temperature 30 °C (We = 0.92, Re = 206.25, Oh = 4.7 × 10^−3^, Ste = 0.722). Evolution of the incoming droplet (the second one) impacting onto a gold-plated glass substrate recorded using a high-speed digital camera with condition D in Table 2. The dashed lines separate the outline of the droplet from that of its reflection on the substrate. The scale bars are 50 μm.

A high-speed digital camera was also used to observe the deposition of molten metal droplets under a stage velocity of 50 mm∙s^−1^ (frequency = 1000 Hz) on a gold-plated glass substrate with condition D in Table 2, as shown in Figure 7. The dot spacing of the droplet was set at 50 μm. The start of a droplet impacting is designated as 0 μs. At 10 μs, the molten droplet impacts onto the substrate and spreads out to its maximum diameter to the side of the solidified droplet on the substrate. In the period 20–30 μs, the droplet comes to the highest point in the vertical direction and is close to the first droplet (solidified droplet). Between 40 and 350 μs, the droplet of molten metal solder sticks to the substrate and starts post-spreading oscillations in a process of recoil with time. Wang et al. [6] examined molten micro droplets on a gold-plated glass substrate, and found that the droplet of a complete solid was determined by the solidification time of the molten droplet at 350 μs. Finally, the droplets were deposited side-by-side on the gold-plated glass substrate. When the velocity of the stage is 50 mm∙s^−1^, and the time interval between the droplets is set at 1 ms, the flying and solidification times of the droplets are 450 and 350 μs, respectively. The results show that the molten micro droplet can be successfully deposited sequentially on the gold-plated glass substrate with a 1 ms time interval.

### 3.3. Study of the Deposition Process by Changing the Substrate Temperature

**Figure 8:**
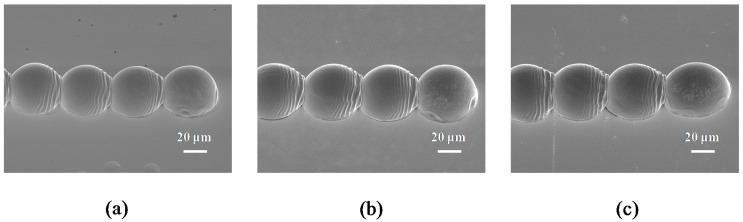
The deposition of a molten metal droplet under different stage temperatures with the following initial characteristics: diameter D_0_ = 50 μm, velocity = 1.1 m∙s^−1^, dot spacing = 50 μm, and motion velocity = 50 mm∙s^−1^ (frequency = 1000 Hz). (**a**) 30 °C (Ste = 0.722); (**b**) 50 °C (Ste = 0.645); (**c**) 70 °C (Ste = 0.567). The scale bars are 20 μm.

Figure 8 shows SEM images of the deposition process at substrate temperatures of 30, 50 and 70 °C, when the droplet temperature was kept constant at 230 °C. Detailed views of the droplet deposition can be seen in the photographs. Li et al. [19] investigated the successive deposition of uniform molten aluminum droplets on a horizontally moving substrate. They found that the L-shaped ridges of the lines on the solidified drop surface are due to alternately spreading and recoiling of the droplet between flow oscillations and solidification. In order to obtain better quantitative agreement from 30 to 70 °C, we measured the average width of the metallic line from SEM photographs during droplet deposition and mergence with the successive droplets. The measured values of the metallic line after the impact are shown in Table 3. In this experiment, the line widths of the droplet deposited at substrate temperatures of 30, 50 and 70 °C were 55, 56 and 59 μm, respectively. The results of the experiment show that good deposition between the droplets was achieved with a suitable combination of sample temperatures, from 30 to 70 °C.

**Figure 9:**
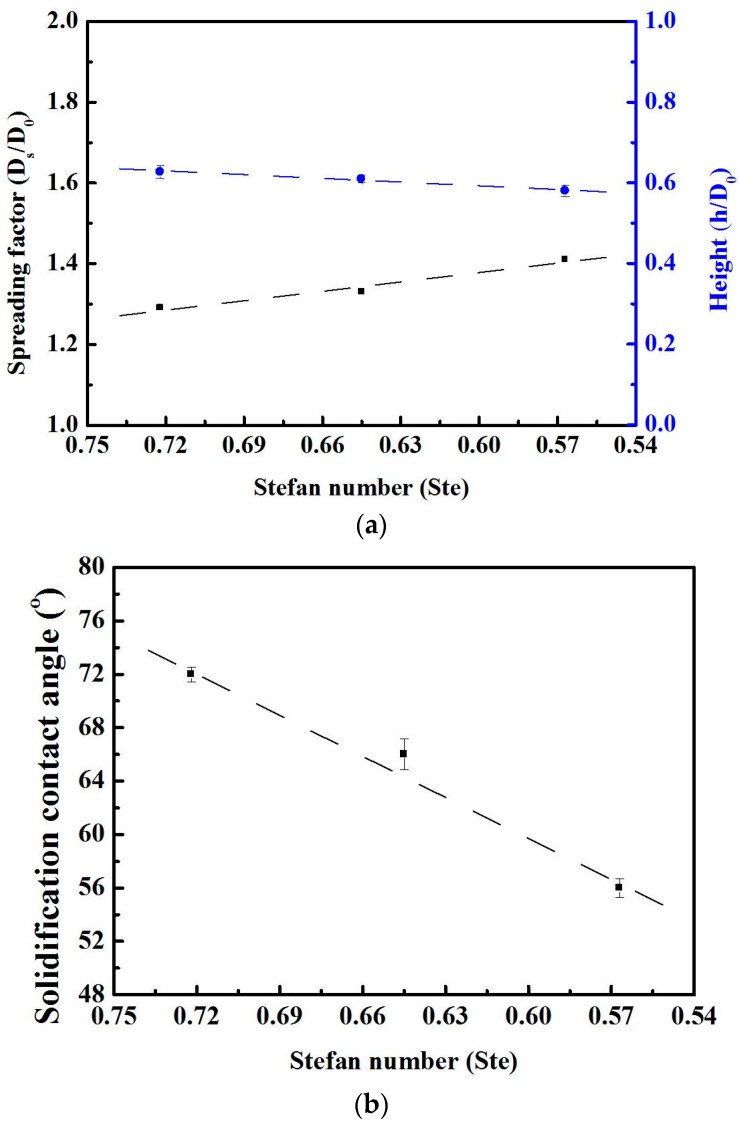
The spreading diameter of a micro metal droplet and the final shape of droplets formed on a gold-plated glass substrate. (**a**) The experimentally determined spread factor and height for the different substrate temperatures (Stefan number); (**b**) the effects of various substrate temperatures on the solidification contact angle of the droplets.

Figure 9 presents the effects of the sample temperature (Stefan number) on the substrate. The deposition of molten micro droplets was examined using the spread factor, height and solidification contact angle. Each data was measured five times and the average value was taken as the final one. The spreading diameters of the droplet deposited at substrate temperatures of 30, 50 and 70 °C were 64.4, 66.7 and 70.6 μm, respectively. The initial diameter (D_0_) of the solder alloy Sn-3Ag-0.5Cu droplet is 50 μm. Each sample was calculated and validated using the spreading factor parameter ζ_max_(t) defined as: [41]

(6) $$\zeta_{\max}(t)=\frac{D_{\max}(t)}{D_{0}},$$

where D_max_(t) is the diameter of the contact disk between the droplet and the substrate and D_0_ is the initial diameter of the droplet before impact.

The heights of the droplet deposited at substrate temperatures of 30, 50 and 70 °C were 30.5, 30.0 and 27.8 μm, respectively. The height (H) of the droplets is defined as follows [42]

(7) $$H=\frac{h}{D_{0}},$$

where h is the impact height of the droplet based on the initial droplet diameter, and D_0_ is the pre-impact initial diameter.

The solidification contact angles of the droplet deposited at substrate temperatures of 30, 50 and 70 °C were 72°, 66° and 56°, respectively. The solidification contact angles between the droplet and substrate were measured by using a contact angle (CA) system (OCA 20, Dataphysics Instruments GmbH, Filderstadt, Germany).

Based on these results, the appropriate spreading factor and height of droplets between 30 and 70 °C were obtained by increasing the sample temperature, and the droplet size of the pre-impact phase was 50 μm. When the sample temperature increased from 30 to 70 °C, the spreading factor increased from 1.29 to 1.41 and the height decreased from 0.63 to 0.58. A linear curve fitted to the height yields the following correlation, plotted in Figure 9a:

Height (h/D_0_) = 0.4079 + 0.30797 × Stefan number, (8)

It is clear that the solidification contact angle decreased from 72° to 56°. The results show that the impact of the droplets formed a steady line, which shows that the solidification contact angle decreases along with the Stefan number, as plotted in Figure 9b:

Solidification contact angle = −1.73748 + 102.40322 × Stefan number,
(9)

Therefore, the height and solidification contact angle decreased with the Stefan number.

### 3.4. Formation of 2D Electronically Conductive Structures

**Figure 10:**
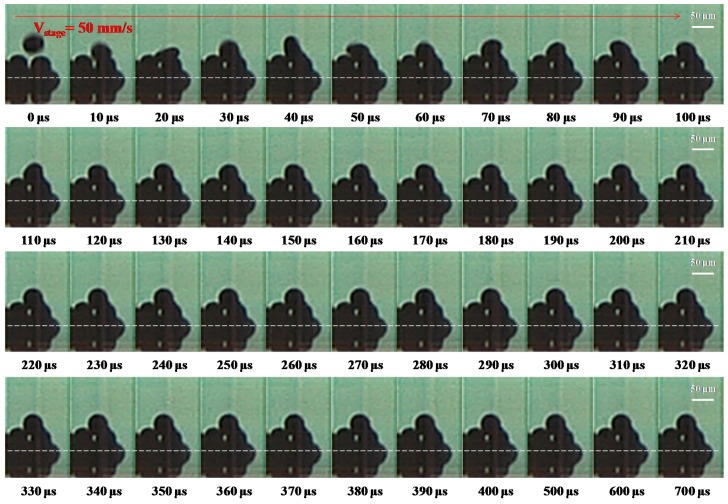
Evolution of the incoming droplet under a dot spacing of 50 μm impacting onto a solidified droplet recorded using a high-speed digital camera with condition E in Table 2. The dashed lines separate the outline of the droplet from that of its reflection on the substrate at 50 °C (Ste = 0.645). The scale bars are 50 μm.

Figure 10 shows high speed camera images of vertical walls fabricated with a dot-spacing of 50 μm at a substrate temperatures of 50 °C with condition E in Table 2. As the stage velocity was set as 50 mm∙s^−1^, the frequency of the droplet ejection was 1000 Hz. The droplets did not merge during their coalescence in the second layer. The incoming droplet impacted and spread the solidified droplet of the second layer from 10 to 20 μs. In the period 30–40 μs, the droplet recoiled on the surface of the solidified droplet. Wang et al. [6] examined the successive behavior of molten micro droplets deposited on a gold-plated glass substrate using inkjet printing techniques, and found that the second (molten) droplet achieved complete solidification at 220 μs. Detailed micro pores of the layers between droplets can be seen by using a high-speed digital camera. Finally, the droplet reached its static state, and the equilibrium stage of the droplet is then reached gradually with a height of 31 μm.

**Figure 11:**
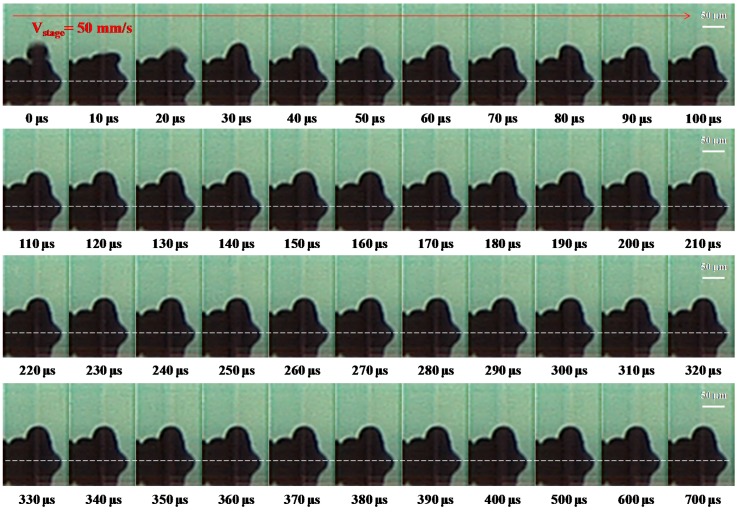
Evolution of the incoming droplet under a dot spacing of 50 μm impacting onto a solidified droplet recorded using a high-speed digital camera with condition F in Table 2. The dashed lines separate the outline of the droplet from that of its reflection onto a substrate at 70 °C (Ste = 0.567). The scale bars are 50 μm.

Figure 11 shows the high-speed camera images of the vertical walls fabricated with a dot-spacing of 50 μm at a substrate temperature of 70 °C with condition F in Table 2. The stage velocity was set at 50 mm∙s^−1^, and the droplet ejection frequency was 1000 Hz, and with this conditions, the layers between droplets had no obvious micro pores. At 10 μs, the incoming droplet impacted the solidified droplet and then spread along its surface. Between 20 and 30 μs, the droplet recoiled back to the highest point. Afterwards, the incoming droplet was deposited and this resulted in a height of 27 μm. As the sample temperature increased to 70 °C, the micro pores at the interface were no longer visible and the droplets deposited successfully in different layers. The wall of the structure in Figure 11 shows good deposition with condition F (the substrate temperature of 70 °C), whereas the droplets in Figure 10 have not merged clearly with condition E (the substrate temperature of 50 °C).

**Figure 12:**
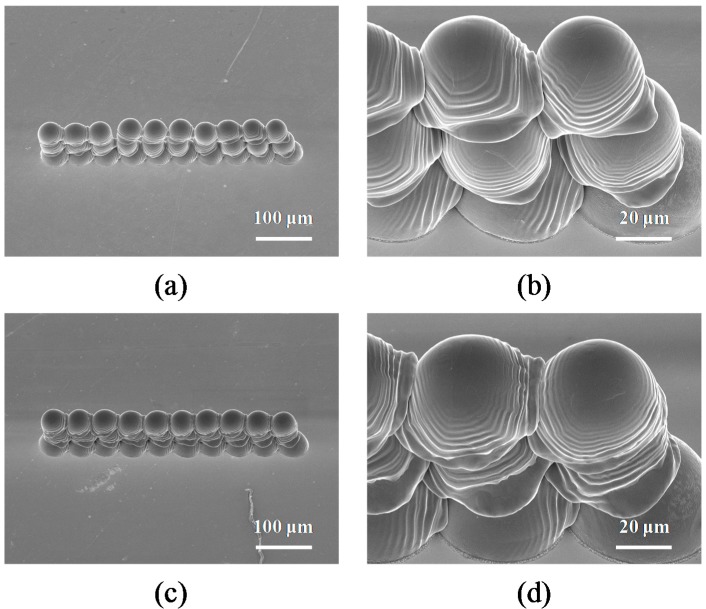
Tilted 40° SEM images of the deposited droplets when the substrate temperature is controlled, and the dot spacing and stage velocity are fixed at 50 μm and 50 mm∙s^−1^ (frequency = 1000 Hz), respectively. (**a**) 50 °C (Ste = 0.645), 200× magnification; (**b**) 50 °C (Ste = 0.645), 1000× magnification; (**c**) 70 °C (Ste = 0.567), 200× magnification; (**d**) 70 °C (Ste = 0.567), 1000× magnification.

Figure 12 shows detailed 40° tilted SEM views of droplets for the vertical walls fabricated with a dot-spacing of 50 μm at substrate temperatures of 50 and 70 °C. It can be observed that the micro pattern forms and deposits onto the gold-plated glass substrate in each cycle of the waveform. Small micro pores between droplets in the adjacent layers can be clearly seen in Figure 12a,b, when the substrate temperature was 50 °C. As the substrate temperature increased to 70 °C, the micro pores between droplets were no longer visible in Figure 12c,d. Therefore, the substrate temperature of 70 °C was identified as the minimum temperature for printing the 3D micro pattern. In this example, the solidification of molten micro droplets occurred as soon as the incoming droplet contacted the solidified droplet. It can be seen that a series of ridges formed rapidly from the solidified droplet into the oscillating liquid. This indicates the spreading and recoiling of the droplet both changed due to the oscillation-induced inertial pressure, and then every overlap of the droplet occurred in the parallel direction. Kim et al. [43] examined the effects of ball size and porosity on the thermophysical properties of Sn-3.0Ag-0.5Cu solder balls, and found that the thermal diffusivity decreased by as much as 28% for the same ball sizes and pressures were measured using a laser flash apparatus over a temperature range from RT to 150 °C. This is a key condition that needs to be considered when choosing the thermal diffusivity and thermal conductivity coefficient of these materials, which may include Sn-3.0Ag-0.5Cu solder and gold. However, the solidification time could be estimated by utilizing a formula, which gives [44]

(10) $$\tau_{s}=\frac{2a^{2}}{3a}\frac{k}{k_{t}}\left(\ln\left(\frac{T_{0}-T_{a}}{T_{f}-T_{a}}\right)+\left(1+\frac{k_{t}}{2k}\right)\frac{L}{c\left(T_{f}-T_{a}\right)}\right),$$

where *a* is the diameter of spreading droplet, *α* is the thermal diffusivity of the droplet, *k* and *k_t_* are the thermal conductivities of the droplet and gold, respectively. T_0_ is the initial temperature of the droplet, *T_a_* is the substrate temperature, T_f_ is the fusion temperature of the droplet, L is the latent heat of fusion, and c is the melt’s specific heat. In this study, T_0_ = 230 °C, T_f_ = 217 °C, L = 64,762 J∙kg^−1^ and c = 250 J∙kg^−1^∙°C ^−1^.

The solidification times of the droplet at substrate temperatures of 50 and 70 °C were 174 and 224 μs, respectively. It was thus found that the solidification time of the molten micro droplets increased as the temperature of the sample rose from 50 to 70 °C. The micro pores of the 3D micro patterns could be improved by increasing the temperature of the sample when inkjet printing on the substrate. Based on these results, it is evident that parallel lines of droplets formed together with no visible micro pores on the interface between droplets at 70 °C.

## 4. Conclusions

In this study we observed and analyzed the deposition of molten micro droplets using the piezoelectric inkjet printing technology. The 3D micro patterns were produced on the gold-plated glass substrates by controlling the dot spacing of the droplets, the stage velocity and the sample temperature. The conclusions of this work are summarized as follows:

1. The metallic line with a width of 55 μm can be successfully printed with respect to the value of the dot spacing (50 μm) and the stage velocity (50 mm∙s^−1^) at the substrate temperature of 30 °C. However, the L-shaped ridges of the lines on the solidified drop surface were formed for the time interval of 1 ms.
2. With regard to the increase in sample temperature (from 30 to 70 °C), the height (from 0.63 to 0.58) and solidification contact angle (from 72° to 56°) of the metallic micro droplets gradually decreased on the substrate.
3. The results of the SEM observations showed that the quality of 3D micro patterns improved significantly at 70 °C. The parallel lines of droplets were formed together with no visible micro pores on the interface between droplets.

## Figures and Tables

**Table 1:** Thermal and physical properties of Sn-3Ag-0.5Cu used in the formula for the experimental calculating [18,39].

Parameters	Value	Unit	Symbol
Density	7500	kg∙m^−3^	ρ
Viscosity	2	mPa∙s	η
Surface tension coefficient	0.431	N∙m^−1^	σ
Liquidus temperature	221	°C	θ_l_
Solidus temperature	216	°C	θ_s_
Specific heat of liquid	250	J∙kg^−1^∙°C^−1^	C_p_
Equilibrium freezing temperature of solder	217	°C	T_m_
Latent heat of fusion	64,762	J∙kg^−1^	L

**Table 2:** The experimental conditions of the inkjet printing.

Condition	Pulse Time (μs)					Drop Frequency (Hz)	Pulse Voltage (V)			Jet Height (mm)	Reservoir Pressure (kPa)	Sample Temperature (°C)
	T_rise_	T_dwell_	T_fall_	T_echo_	T_finalrise_		V_DC_	V_1_	V_2_			
A	200	20	2	6	6	200	−40	−35	−50	0.5	1.0	30
B	200	20	2	6	6	100	−40	−35	−50	0.5	1.0	30
C	200	20	2	6	6	50	−40	−35	−50	0.5	1.0	30
D	200	20	2	6	6	1000	−40	−35	−50	0.5	1.0	30
E	200	20	2	6	6	1000	−40	−35	−50	0.5	1.0	50
F	200	20	2	6	6	1000	−40	−35	−50	0.5	1.0	70

**Table 3:** Comparison of sample conditions based on the results of the experiment and related calculation formula.

Sample Temperature (°C)	Weber Number	Reynolds Number	Ohnesorge Number	Stefan Number	The Average Width of Metallic Line (μm)
30	0.92	206.25	4.7 × 10^−3^	0.722	55 ± 1.3
50	0.92	206.25	4.7 × 10^−3^	0.645	56 ± 1.4
70	0.92	206.25	4.7 × 10^−3^	0.567	59 ± 1.7
